# Comparative Physiological and Metabolic Analysis Reveals a Complex Mechanism Involved in Drought Tolerance in Chickpea (*Cicer arietinum L*.) Induced by PGPR and PGRs

**DOI:** 10.1038/s41598-019-38702-8

**Published:** 2019-02-14

**Authors:** Naeem Khan, Asghari Bano, M. Atikur Rahman, Jia Guo, Zhiyu Kang, Md. Ali Babar

**Affiliations:** 1 Department of Plant Sciences, Quaid-I-Azam University, Islamabad, Pakistan; 2 Department of Biosciences, University of Wah, Wah Cantt, Pakistan; 3 Department of Agronomy, IFAS, University of Florida, Gainesville, FL USA; 4 Zhiyu Kang, College of Agronomy and Biotechnology, Yunnan Agricultural University, Kunming, Yunnan province (650201) China

## Abstract

The plant growth promoting rhizobacteria (PGPR) and plant growth regulators (PGRs) can be applied to improve the growth and productivity of plants, with potential to be used for genetic improvement of drought tolerance. However, for genetic improvement to be achieved, a solid understanding of the physiological and biochemical changes in plants induced by PGPR and PGR is required. The present study was carried out to investigate the role of PGPR and PGRs on the physiology and biochemical changes in chickpea grown under drought stress conditions and their association with drought tolerance. The PGPR, isolated from the rhizosphere of chickpea, were characterized on the basis of colony morphology and biochemical characters. They were also screened for the production of indole-3-acetic acid (IAA), hydrogen cyanide (HCN), ammonia (NH_3_), and exopolysaccharides (EPS) production. The isolated PGPR strains, named P1, P2, and P3, were identified by *16S-rRNA* gene sequencing as *Bacillus subtilis*, *Bacillus thuringiensis*, and *Bacillus megaterium*, respectively. The seeds of two chickpea varieties, Punjab Noor-2009 (drought sensitive) and 93127 (drought tolerant) were soaked for 2–3 h prior to sowing in 24 h old cultures of isolates. The salicylic acid (SA) and putrescine (Put) were sprayed (150 mg/L) on 25 day old chickpea seedlings. The results showed that chickpea plants treated with a consortium of PGPR and PGRs significantly enhanced the chlorophyll, protein, and sugar contents compared to irrigated and drought conditions. Leaf proline content, lipid peroxidation, and activities of antioxidant enzymes (CAT, APOX, POD, and SOD) all increased in response to drought stress but decreased due to the PGPR and PGRs treatment. An ultrahigh performance liquid chromatography-high resolution mass spectrometry (UPLC-HRMS) analysis was carried out for metabolic profiling of chickpea leaves planted under controlled (well-irrigated), drought, and consortium (drought plus PGPR and PGRs) conditions. Proline, L-arginine, L-histidine, L-isoleucine, and tryptophan were accumulated in the leaves of chickpea exposed to drought stress. Consortium of PGPR and PGRs induced significant accumulation of riboflavin, L-asparagine, aspartate, glycerol, nicotinamide, and 3-hydroxy-3-methyglutarate in the leaves of chickpea. The drought sensitive chickpea variety showed significant accumulation of nicotinamide and 4-hydroxy-methylglycine in PGPR and PGR treated plants at both time points (44 and 60 days) as compared to non-inoculated drought plants. Additionally, arginine accumulation was also enhanced in the leaves of the sensitive variety under drought conditions. Metabolic changes as a result of drought and consortium conditions highlighted pools of metabolites that affect the metabolic and physiological adjustments in chickpea that reduce drought impacts.

## Introduction

Though plants are often exposed to many different types of stresses^1^, abiotic stresses are responsible for most of the major losses in crop productivity^2–4^. Among the abiotic stresses effecting crop productivity, drought is the most important environmental stress. Drought has been shown to limit plant growth, distribution, and yield^5–7^ and has become a serious problem in global food security^8^. Moreover, the current climate change trends are expected to have a major impact on precipitation profiles, increasing the occurrence and intensity of drought around the world. There is an urgent need to enhance drought tolerance in pulses in order to improve their growth and yield^9^. Chickpea ranked third among pulses after peas and soybean, with a total of 15% of the world’s pulse production belonging to this crop^10^. Chickpea is the principle source of vegetable protein in Pakistan and occupies 73% of the total area under pulse cultivation. Drought stress in rainfed areas is a major limiting factor that affects crop production per hectare. Yield losses due to drought stress range from 15–60% depending on the geographical condition of the area and the length of the dry spell. Hence, there is a critical need to increase drought tolerance in legumes^9^. Thus, strategies may be developed to manage drought stress and to develop drought tolerance in crop plants^11^. Recent studies suggest that PGPR along with PGRs can help plants to cope with drought stresses^12^.

Bacteria that colonize plant roots and encourage plant growth are denoted as plant growth-promoting rhizobacteria (PGPR). PGPR have significant impacts on plant growth and development as they improve the availability of micro-nutrients to their host plant by assembly of growth promoting chemicals. They are also well-known for their role in improving growth patterns of roots. A huge diversity of organic compounds ooze out from the roots as exudates that act as a signal for attracting soil microbes as they are a rich source of carbon supply within the soil^13,14^. Rhizo-sheath formation around the roots is the function of a class of soil saccharides known as exopolysaccharides^15^. Exopolysaccharides (EPS) are large carbohydrates usually synthesized and released in the rhizosphere by soil microorganisms^16^. EPS play a key role in protecting the plant from desiccation^17^ and from antimicrobial activity of various predators^18^. It also helps in maintaining primary cellular functions^15,18^. Soil bacteria maintains mutualistic interactions with plant roots that enable plants to grow well and tolerate several abiotic stresses, including drought, salt, heavy metals, and pathogens^19,20^. It has been previously reported that PGPR can enhance tolerance of crops to various abiotic stresses by improving the level of cellular metabolites, which suggests a novel role of PGPRs to interact with plant metabolomes as well as to influence the plant microbiome^21,22^. These bacteria are also responsible for the up- or down-regulation of various genes linked with plant metabolites under abiotic stresses. This indicates an alteration in plant metabolic expression patterns under drought stress conditions which may lead to drought tolerance in crop plants^23^.

Plant growth regulators (PGRs) are chemical compounds that significantly affect the growth and differentiation of plant cells and tissues^24^. They act as messengers for intercellular communication^25^. They have been associated with the control of biotic and abiotic stresses as well as upholding water conservation status in plants^26^. Salicylic acid (SA), a phenolic compound, is involved in the regulation of growth and development of plants and their responses to drought stress^27^. It is evident that SA provides protection in plants against abiotic stresses by regulating important physiological processes such as photosynthesis, proline metabolism, antioxidant defense system, and plant-water relations^27–29^. War *et al*.^30^ demonstrated the role of SA in physiological, morphological, and biochemical mechanisms of chickpea. The beneficial effects of SA on growth, flowering, and flavonoid production in ornamental and crop plants had also been reported previously^31^. SA functions as a signalling compound under stress which induces genes that function as chaperones, antioxidant enzymes, and heat shock proteins, as well as genes responsible for the synthesis of secondary metabolites^32^. The naturally occurring diamine and putrescine have been assumed as latent regulators of plant growth and water conservation and also encourage plant root development^33^. The plant growth regulators are known for their major role in mediating plant defense responses against abiotic stresses and pathogen attacks as they intricate with metabolic expression of plants under stress. These PGRs are also involved in the process of stomatal closure, thus maintaining water balance and regulating stress responsive genes. They also intricate with the processes of plant development, flowering, fruiting, ripening, senescence, and expression of secondary metabolites associated with drought tolerance of plants^27,34^.

Both PGPRs and PGRs exert beneficial effects on plant growth when applied alone, however, their combined applications were more effective than PGPR or PGRs used alone to mitigate drought stress in wheat and chickpea^35,36^. Our prior study^35^ clearly demonstrated that the addition of PGRs to PGPR inoculated plants significantly enhanced the leaf chlorophyll and sugar content, and assisted in osmoregulation and ameliorated oxidative stresses and induced new proteins. Combined application of PGRs and PGPR decreased lipid peroxidation more effectively and increased the leaf area. The relative water content in leaves, and root fresh and dry weight were also higher in combined treatment of PGPR and PGRs. The nutrient content of rhizosphere soil of PGPR and PGRs treated plants was also enhanced significantly as compared to single application of PGPR and PGRs. It is inferred from our previous studies that PGPR and PGRs work synergistically to promote growth of plants under moisture and nutrient deficit condition^21,35,36^. To use PGPR and PGRs for genetic improvement of drought tolerance in chickpea, we need to have a clear understanding of the biochemical processes involved with different physiological mechanisms and their relationships to different traits. Untargeted ultrahigh-performance liquid chromatography-high resolution mass spectrometry (UPLC-HRMS) based metabolic profiling is a powerful tool used for broad spectrum identification and quantification of metabolites in plants. Metabolome profiling is one of the most current and popular techniques to be used over the last decade. Metabolic markers attract researchers due to their close association with phenotypic characters^37,38^. Some metabolites have been described to directly intricate in the tolerance of plants^39^.

Drought has significant effects on the metabolic changes in plants^21,38,40^. In our earlier study on chickpea under drought stress conditions, we reported significant differences in metabolite accumulation under drought stress conditions in two varieties contrasting for drought tolerance^21^. The most pronounced increase in metabolite accumulation due to drought stress was demonstrated for allantoin, L‐proline, L‐arginine, L‐histidine, L‐isoleucine, and tryptophan. Corresponding to those metabolic changes, varieties also showed differences in different physiological and biochemical traits including antioxidant activities. Some other studies also demonstrated that pools of metabolites intricate in the physiology and metabolism of crop plants during severe stress conditions^41,42^. Sugars, amino acids, organic acids, and polyols play a key role in drought tolerance^43^. Amino acids and organic acids are responsible for the maintenance of water potential gradients from soil to plants, while sugars are involved in osmotic adjustment^40,44^. However, the information on comparative analysis of physiological traits and metabolic profiling affected by PGPR and PGRs application is not available in chickpea. The comparative analysis identifies the key metabolites that are differentially accumulated between drought sensitive and tolerant chickpea varieties under PGPR + PGRs treatment. Thus, the present study was aimed to evaluate the effect of PGPR and PGRs (SA and Put) consortium on the physiology of chickpea grown in drought stress conditions and to correlate the metabolic profiling in leaves of chickpea exposed to drought stress and treated with the PGPR and PGRs consortium.

## Results

### Phosphorus Solubilization Index

The three isolated plant growth-promoting rhizobacteria: *Bacillus subtilis*, *Bacillus thuringiensis*, and *Bacillus megaterium*, were phosphate solubilizers (Table 1). *Bacillus subtilis* was the most effective phosphorus solubilizer with a phosphorus solubilization index of 2.822 followed by *Bacillus megaterium* with a solubilization index of 2.621.

**Table 1:** Proline, IAA, HCN, NH_3_ production and P-solubilisation index by Selected PGPR strains.

Selected PGPR Strains	Proline (μg/mg)	IAA (μg/ml)	HCN production		NH_3_	PSI
			Qualitative	Quantitative		
*B. subtilis*	1.011 c	0.499 a	−	0.011 c	+	2.822 a
*B. thuringiensis*	1.699 a	0.442 ab	++	0.082 b	+	2.411 c
*B. megaterium*	1.671 ab	0.381 c	+++	0.097 a	+	2.621 ab

HCN production (based on intensity of color): −negative, + weak, + + moderate, + + + strong, PSI = Phosphate solubilisation index.

### Proline, IAA, HCN, and NH_3_ Production by Selected PGPR Isolates

Maximum proline production (1.699 μg/mg) was recorded in *Bacillus thuringiensis*, followed by *Bacillus megaterium* (1.671 μg/mg) (Table 1). Except for *B. subtilis*, the 2 selected PGPR strains indicated the presence of hydrogen cyanide. *Bacillus megaterium* was found to be most effective with maximum O.D value of 0.097, followed by *Bacillus thuringiensis* (0.082). *Bacillus subtilis* was the most effective strain for IAA production, followed by *Bacillus thuringiensis*. All the strains were found to be positive for NH_3_ production.

### Antibacterial and Antifungal Activities of PGPR

Bacillus subtilis, Bacillus thuringiensis, and Bacillus megaterium inhibited the growth of Staphylococcus aureus, Pseudomonas aeruginosa, Klebsiella pneumonia, and Escherichia coli (Table 2). Maximum inhibition (63%) in mycelial growth of Helminthosporium sativum was recorded due to Bacillus subtilis. Bacillus thuringiensis significantly suppressed the growth of Fusarium solani (81%) (Table 2).

**Table 2:** Antibacterial and Antifungal Activities of Selected PGPR Strains.

Antibacterial Activities					Antifungal Activities	
PGPR Strains	*Staphylococcus aureus*	*Pseudomonas aeruginosa*	*Klebsiella pneumoniae*	*Escherichia coli*	*Helminthosporium sativum*	*Fusarium solani*
*B. subtilis*	+	+	+	+	63%	78%
*B. thuringiensis*	+	+	+	+	43%	81%
*B. megaterium*	+	+	+	+	56%	71%

+positive.

### Chemical Composition of Exopolysaccharides (EPS) Produced by PGPR

The lyophilized EPS was soluble in water and insoluble in benzene, acetone, and chloroform (Table 3). The sugar and protein contents of EPS were 96% and 98% in *Bacillus megaterium*, 95% and 98% in *Bacillus subtilis and Bacillus thuringiensis*. *Bacillus megaterium* exhibited maximum (94%) uronic acid.

**Table 3:** Chemical Characterization of Exopolysaccharides (EPS).

PGPR Strains	Sugar content (μg/g)	Protein content (μg/g)	Uronic acid (μg/mg)
Control	221.3 d	11.2 d	0.062 c
*Bacillus subtilis*	4983.7 b	592.5 c	0.91 b
*Bacillus thuringiensis*	4354.1 c	639.7 a	1.04 ab
*Bacillus megaterium*	5521.4 a	623.3 ab	1.13 a

### Alignment of *16S rRNA* Sequence

For the strain P1, isolated from the rhizosphere of chickpea (at Karak, with 7% soil moisture content), a total length of sequence with 1557 nucleotides was obtained. The evaluation of the nucleotide sequence of isolate P1 with data nucleotide bank revealed 100% (1506/1506 bases) similarity with *Bacillus subtilis* (Accession No. MF616407). For the strain P2, isolated from the rhizosphere of chickpea (at Bhakkar, 6% soil moisture content), the total length of sequence with 1517 nucleotides was obtained. The evaluation of the nucleotide sequence with data nucleotide bank indicated sequence similarity of 99% (1514/1517 bases) with *Bacillus thuringiensis* (Accession No. MF662971). For the strain P3, isolated from the rhizosphere of chickpea (at Cholistan, 4% soil moisture content), the total length of sequence with 1474 nucleotides was obtained. The evaluation of the nucleotide sequence with data nucleotide bank showed maximum sequence similarity of 99% (1492/1498 bases) with *Bacillus megaterium* (Accession No. MF008110)^35^.

### Leaf Chlorophyll Content and Chlorophyll Fluorescence

**Figure 1:**
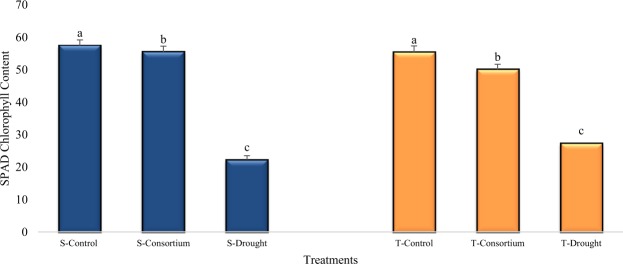
Mean chlorophyll content (±SE) in the leaves of drought sensitive (S) and tolerant variety (T) varieties under irrigated, drought, and consortium treatments across 14 and 25 days after water treatment imposition. Data are means of six replicates along with standard error bars. Different letters are indicating significant differences (P < 0.05) among treatments (control vs consortium vs drought) for a variety.

In general, the chlorophyll content decreased significantly in both the sensitive (S) and tolerant varieties (T) under drought condition as compared to irrigated control (Fig. 1). However, the percent decrease in the plants treated with PGPR + PGRs consortium was lower as compared to untreated plants exposed to drought stress. The drought stress decreased the chlorophyll content by 61% in the sensitive variety and 42% in the tolerant variety but the consortium of PGPR + PGR reduced the percent damage to 4% and 9% in the sensitive and tolerant varieties, respectively, as compared to the control. The photochemical efficiency of photosystem II (*F*v/*F*m) showed variability between varieties grown under stress condition (Table 4). The *F*v/*F*m ratio was decreased for both varieties under the drought condition, but the decrease was more pronounced (64%) for the sensitive variety than for the tolerant variety (26%). The consortium of PGPR/PGRs was most effective in reducing the stress induced decrease in both varieties, with the damage being reduced to (16%) in the drought sensitive variety, whereas the values for the drought tolerant variety were very close to that of irrigated control.

**Table 4:** Mean chlorophyll flourescence (Fv/Fm ratio ± SE) and relative water content (RWC ± SE) of two chickpea varieties under control and drought condition at 14 and 25 days after stress imposition.

Chickpea variety	Chlorophyll flourescence (Fv/Fm)			RWC (%)		
	Control	Consortium	Drought stress	Control	Consortium	Drought stress
Punjab Noor-2009 (Sensitive variety)	0.933 ± 0.011	0.783 ± 0.09	0.336 ± 0.019	78 ± 0.06	61 ± 0.015	25 ± 0.05
93127 (Tolerant variety)	0.921 ± 0.014	0.892 ± 0.008	0.681 ± 0.021	86 ± 0.013	76 ± 0.012	51 ± 0.013

Control = Untreated Irrigated plants; Consortium = PGPR + PGR treated plants; Drought = Untreated plants grown under drought stress.

### Relative Water Content (RWC)

Drought stress caused significant changes in relative water content of both the varieties. The RWC of the sensitive variety (S) decreased by 68% under drought stress as compared to the irrigated control; the tolerant variety (T) showed greater RWC over the sensitive variety. The consortium of PGPR + PGRs significantly reduced the % damage in both of the varieties grown under stress conditions. The PGPR/PGRs treatment significantly enhanced the RWC (59%) in the drought sensitive variety as compared to stress control plants. The PGPR/PGRs treatment also reduced the damage to 21% in the sensitive variety as compared to the irrigated control, whereas in the tolerant variety the damage was reduced to 11% (Table 4).

### Shoot and Root Dry Weights (g)

**Figure 2:**
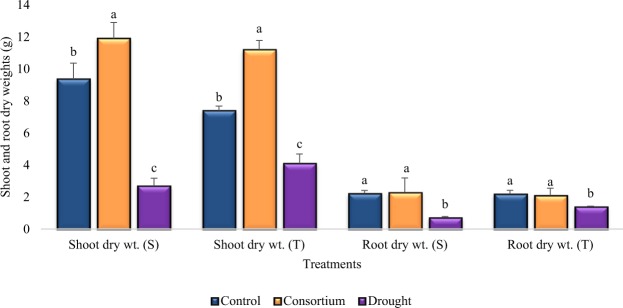
Mean shoot and root dry weights (±SE) of chickpea under control, drought, and consortium treatments after 25 days of drought stress imposition. S-drought sensitive variety (Punjab Noor-2009), T-drought tolerant variety (93127). Error bars represent standard errors of the mean (n = 6). Different letters are indicating significant differences (*P* < 0.05) among treatments (control vs drought vs consortium) for a variety.

Shoot and root dry weights of both the sensitive and tolerant varieties were reduced significantly (*p* < 0.01) due to drought stress as compared to the irrigated control (Fig. 2). The reduction was more pronounced in the sensitive variety than in the tolerant variety. The sensitive and tolerant varieties showed 71% and 45% reduction in shoot dry weight under drought stress conditions compared to irrigated control (Fig. 2). Similarly, 68% and 36% reduction in root dry weights were shown by the sensitive and the tolerant variety, respectively, under drought stress as compared to the control. However, the percent reduction due to drought stress in shoot and root dry weights were controlled by the application of PGPR + PGRs consortium. The PGPR + PGRs consortium treated plants showed significant increases in shoot dry weight as compared to the irrigated control but the root dry weight was at par with the control, even under drought stress conditions.

### Lipid Peroxidation and Antioxidant Enzymes Activity

**Figure 3:**
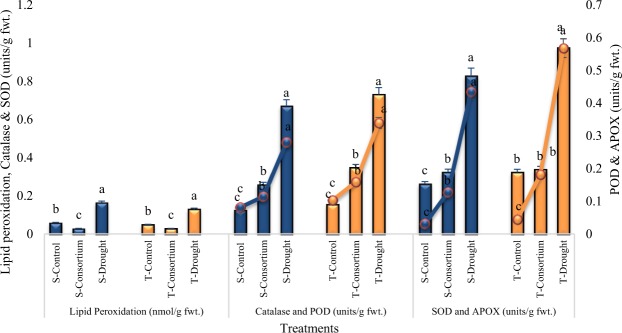
Mean lipid peroxidation (nmol/g fwt.) and antioxidant enzyme activities (±SE) in the leaves of drought sensitive (S) and tolerant variety (T) varieties under irrigated, drought, and consortium treatments. Data are means of six replicates along with standard error bars. Different letters are indicating significant differences (P < 0.05) among treatments (control vs consortium vs drought) for a variety.

The lipid peroxidation was significantly enhanced in plants under drought stress. The application of PGPR + PGRs consortium significantly reduced (82% and 77%) the lipid peroxidation content as compared to the drought stress and untreated control treatments. The consortium of PGPR + PGRs was more effective in the sensitive variety than in the tolerant variety (Fig. 3).

The antioxidant enzyme activities were significantly enhanced in plants under drought stress (Fig. 3). The maximum increases in the sensitive and tolerant varieties, respectively, for ascorbate peroxidase (APOX) activity (93% and 92%) followed by catalase activity (81% and 79%) in the leaves of drought stressed plants as compared to the control were noted. Both the catalase and APOX activities were significantly increased under drought stress over the control and PGPR + PGRs consortium. The tolerant variety showed higher antioxidant activies than the sensitive variety. The antioxidant activities were significantly reduced in plants inoculated with PGPR + PGRs consortium. The most significant decrease (70% and 68%) was noted in ascorbate peroxidase and superoxide dismutase (61% and 65%) activity in both the varieties.

### Leaf Proline, Protein and Sugar Contents

**Figure 4:**
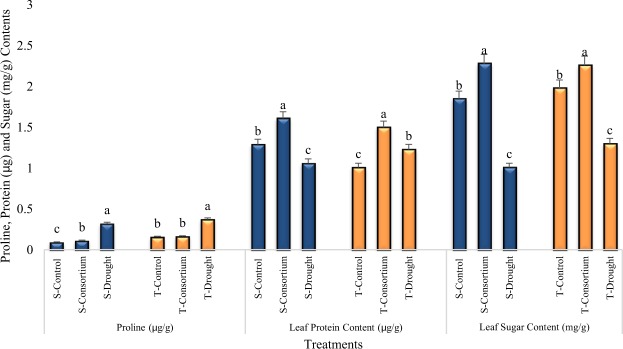
Mean leaf proline, protein and sugar contents (±SE) in the leaves of drought sensitive (S) and tolerant (T) varieties under irrigated, drought, and consortium treatments. Data are average of six replicates along with standard error bars. Different letters are indicating significant differences (P < 0.05) among treatments (control vs consortium vs drought) for a variety.

The proline content significantly increased in plants under drought stress. The tolerant variety showed a higher increase (14%) in proline content than the sensitive variety (Fig. 4). Plants inoculated with PGPR + PGRs consortium had proline production similar to the untreated and irrigated control.

Drought stress caused significant (*p* < 0.01) changes in leaf protein content in both varieties (Fig. 4). The leaf protein content of the sensitive variety (S) decreased by 18% under drought stress as compared to irrigated control; however, the tolerant variety (T) showed greater protein content over the control. The consortium of PGPR + PGRs significantly enhanced the leaf protein content and the increase was even greater (20% and 33%) than control for both the sensitive (20%) and tolerant varieties (33%), respectively. The consortium of PGPR + PGRs significantly enhanced the leaf sugar content as compared to control and drought stress plants (Fig. 4). Drought stress reduced the leaf sugar content significantly by 45% and 34%, respectively, in sensitive and tolerant varieties as compared to control. The Application of PGPR + PGRs consortium on plants grown under drought stress enhanced the leaf sugar content by 56% and 44% in sensitive and tolerant varieties as compared to plants grown under drought stress and 19% and 12% as compared to control plants.

### Profiling of Leaf Metabolites

**Figure 5:**
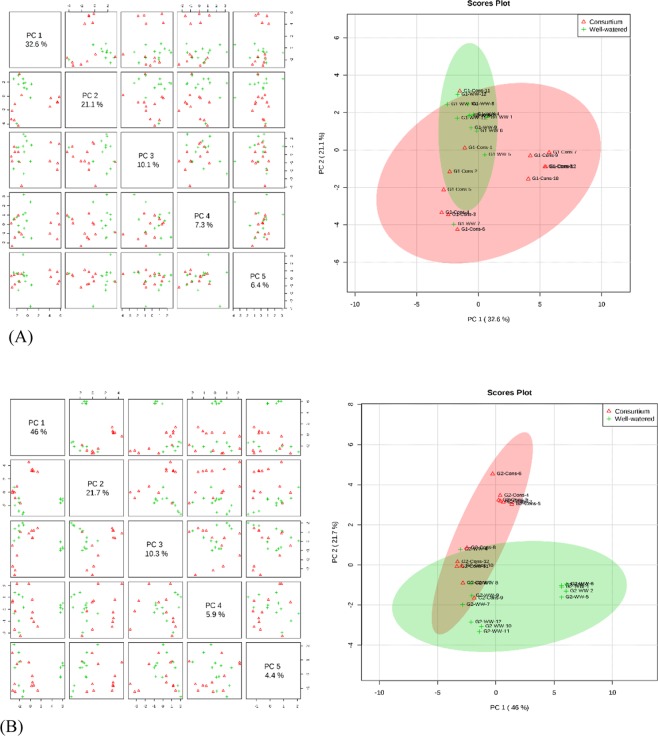
Partial least square discriminant analysis (PLS-DA) and 2D Scores loading plot for the drought sensitive variety Punjab Noor-2009 (**A**) and drought tolerant variety 93127 (**B**) at 2 time points under control (well-watered) and consortium treatments (**A**). Metabolites at two treatments overlapped with each other indicating an unchanged state of metabolite levels in the chickpea leaves.

**Figure 6:**
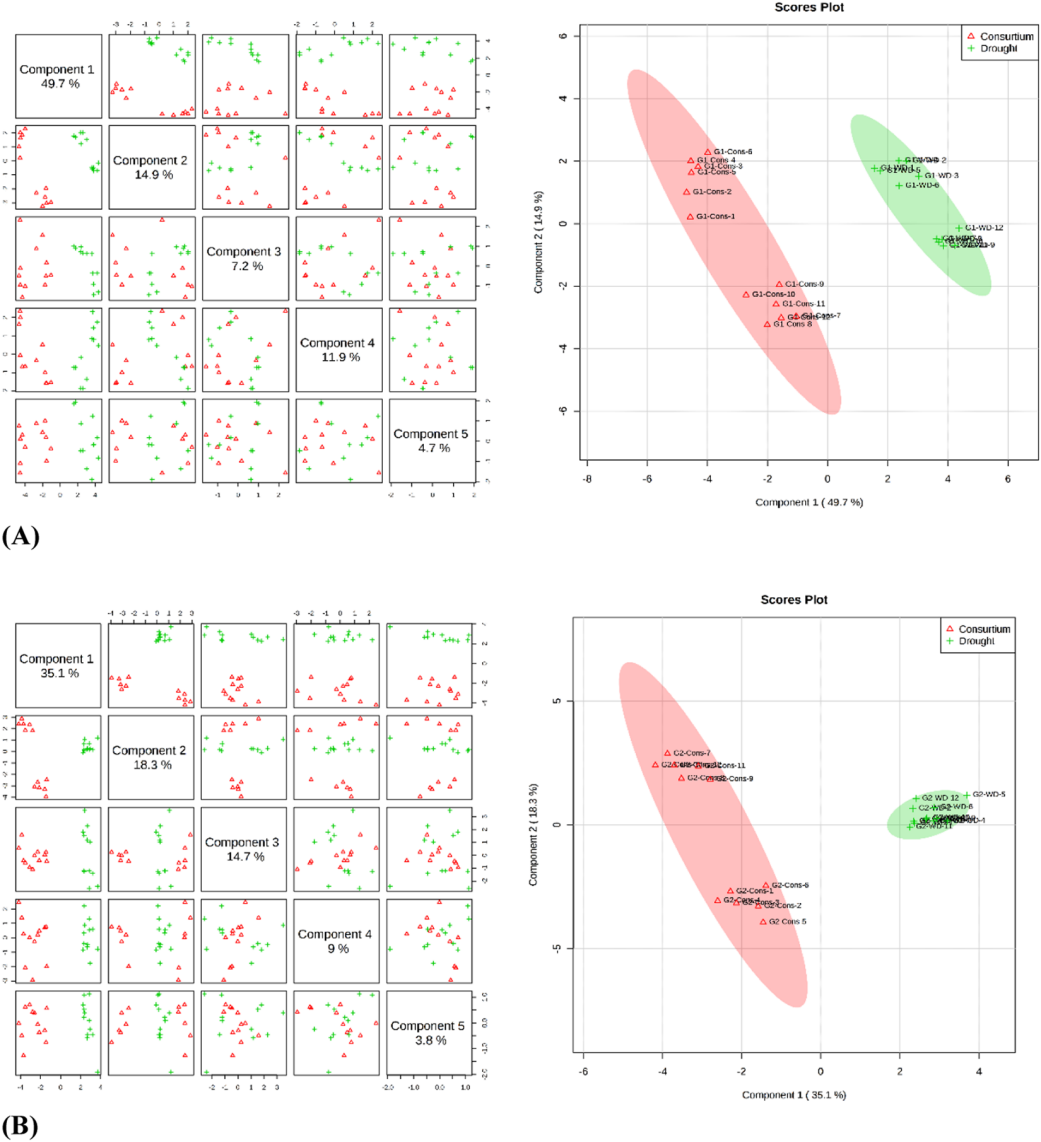
Partial least square discriminant analysis (PLS-DA) and 2 Scores loading plot for the drought sensitive variety Punjab Noor-2009 (**A**) and drought tolerant variety 93127 (**B**) at 2 time points under consortium and drought treatments. Metabolites at consortium and drought treatments did not overlap indicating an altered state of metabolite levels in the chickpea leaves.

The supervised clustering method, Partial Least Squares-Discriminant Analysis (PLS-DA), was performed for both the varieties and for consortium versus control and consortium versus drought treatments. PLS-components (PCs) analysis revealed that component 1 explained 32.6% and 46% of the total variation of the sensitive and tolerant varieties, respectively, under consortium versus control treatment; while the second component explained 21.1% and 21.7% of the variation for sensitive and tolerant genotypes, respectively, for the same treatment. Consortium versus control samples had overlapped in the scores plot between component 1 and 2 for the varieties, which suggests similarity in the metabolic expression in consortium and control samples (Fig. 5A,B). The PLS-DA analysis was also performed for consortium versus drought treatments for both varieties. The first PLS component (PC1) explained 49.7% and 35.1% of the variation across the data sets for both the sensitive and tolerant varieties, respectively. Consortium and drought samples were clearly separated in the scores plot between component 1 and component 2 with no overlapping between the groups (Fig. 6A,B). This distinction of consortium and drought samples clearly indicates the role of PGPR + PGRs consortium in altering the state of metabolites in the leaves of chickpea when exposed to drought stress.

**Figure 7:**
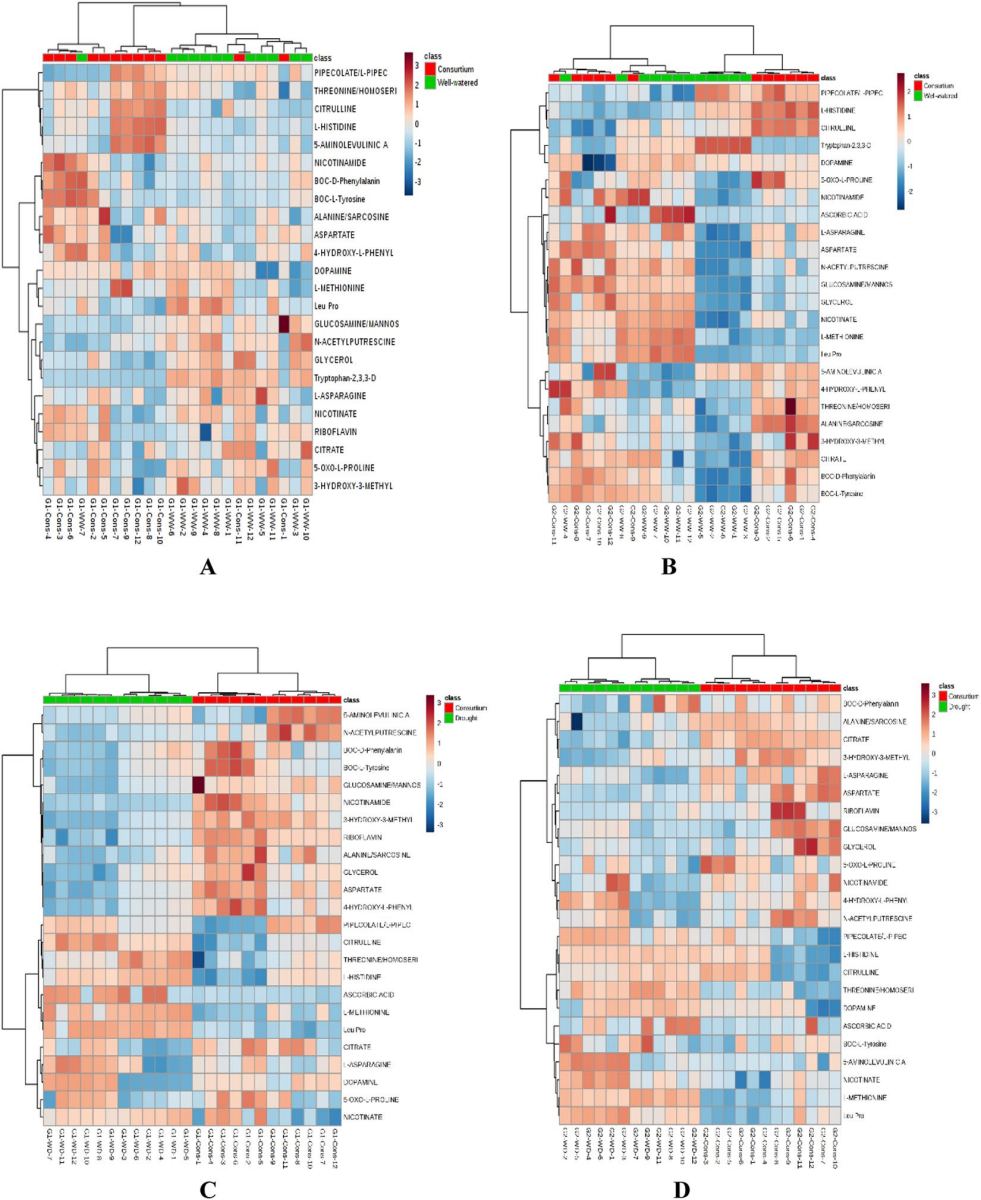
Heat maps, (**A**) (consortium vs well-watered for drought sensitive variety Punjab Noor-2009), (**B**) (consortium vs well-watered for drought tolerant variety 93127), (**C**) (consortium vs drought for drought sensitive variety Punjab Noor-2009), and (**D**) (consortium vs drought for drought tolerant variety 93127), are illustrating top metabolites at two time points. The heat maps were generated based on using Pearson and Ward for distance measure and clustering algorithm, respectively. Metabolite feature areas were normalized and range-scaled across all experimental samples at two different time points.

A total of 55 metabolites were identified through a multi-factorial ANOVA, which were significantly altered in two varieties across time points and treatments (Supplemental Table S1). Among different groups of metabolites, amino acids, vitamins, and other compounds were significantly accumulated in the leaves of plants treated with PGPR + PGRs under drought stress. Amino acids, asparagine, aspartate, Vitamins, riboflavin, nicotinamide and other compounds including glycerol, 4-hydroxy-L-phenylglycine, and 3-hydroxy-3-methylglutarate showed increased levels of accumulation in the leaves of plants treated with PGPR and PGR. Compounds like pipecolate, citrulline, methionine, leucyl proline, and nicotine were highly accumulated in the leaves of untreated plants grown under stress condition (Table S1 and Fig. 7A,B). Significantly different metabolites were analyzed by hierarchical clustering with a heat map in order to visualize the effect of PGPR + PGRs consortium on metabolomics expression over uninoculated irrigated control and uninoculated drought stress plants (Fig. 7A,B). The heat map was generated for consortium versus control and consortium versus drought treatments for both the sensitive and tolerant varieties. The heat map indicates a relationship between metabolites of consortium and control treatments and that the metabolites of the two treatments are intermingled. Pipecolate, threonine, citrulline, and histidine accumulation was higher in the sensitive variety when treated with PGPR + PGRs consortium, whereas hisitidine, citrulline, and alanine accumulation was higher in the tolerant variety treated with PGPR + PGRs consortium compared to control plants. The heat map for the consortium versus drought treatments was divided into two major clusters with a different pattern of metabolite accumulation. The first cluster was represented by metabolites accumulated at higher levels in the leaves of PGPR + PGRs treated (consortium) plants including riboflavin, L-asparagine, aspartate, glycerol, nicotinamide, and 3-hydroxy-3-methyglutarate; whereas, pipecolate, L-methionine, Leucyl proline (leu pro), citrulline, threonine, and nicotinate were abundantly present in leaves of plants grown under drought stress. Accumulation of phenylalanine occurred in both the sensitive and tolerant varieties when plants were exposed to drought stress as well as in PGPR + PGRs treated plants. The sensitive variety showed significant accumulation of nicotinamide and 4-hydroxy-methylglycine in PGPR + PGRs treated plants over uninoculated drought stress plants, whereas the tolerant variety showed similar accumulation only under drought condition. Citrate accumulated equally in both PGPR + PGRs treated plants and uninoculated drought stress plants; however, its accumulation was increased in the tolerant variety treated with PGPR + PGRs consortium. Similarly, alanine and glucosamine concentration was enhanced in both the sensitive and tolerant varieties treated with PGPR + PGRs consortium. The sensitive variety showed increased accumulation of 5-aminolevulinic acid, but its concentration was decreased with the increase in drought period in the tolerant variety. The sensitive variety accumulated N-acetyl putrescine when exposed to drought stress; however, its accumulation was decreased in PGPR + PGRs treated plants. Similar results for N-acetyl putrescine were also reported in the tolerant variety. The PGPR + PGRs treated sensitive variety showed increased levels of dopamine accumulation but reduction in its accumulation occurred with increases in drought period. Increased accumulation of ascorbic acid was reported in the sensitive variety when plants were exposed to drought stress; however, its accumulation decreased in the tolerant variety when treated with PGPR + PGRs consortium or grown under drought stress conditions. When treated with PGPR + PGRs consortium, the tolerant variety accumulated higher levels of citrate, aspartate, alanine, and oxo-proline as compared to the sensitive variety. The clustering of metabolites into two groups clearly indicates the metabolic changes in leaves of PGPR + PGRs treated plants grown under drought condition.

A comparison of statistical models was carried out to identify the important metabolites associated with drought tolerance in plants grown in drought stress and inoculated with PGPR + PGRs consortium by using SAM, PLS-DA, and RF (Table 5 & S1). The SAM plot identified 15 significantly different compounds with the delta value of 1.6, FDR of 0.001 and with less than one (0.3) false positive. Similarly, the most important metabolites were also identified by PLS-DA method based on the VIP score using a five-component model. Random forest classification ranked the important metabolites in order of decreasing prediction accuracy (Mean Decrease Accuracy) using 5000 trees (permutation) with an overall (OOB, out-of-bag) error of 0.0833. Overall, the results were quite similar across all three methods. The topmost important 15 metabolites which were identified by at least 2 different methods are shown in Table 5. These important metabolites include different amino acids, vitamins, sugars, organic acids, amines, fatty acids, and more. The tolerant line demonstrated significantly higher accumulation of leu pro, glycerol, riboflavin, 3-hydroxy-3-methylglutarate, 5-oxo-L-proline, aspartate, and L-methionine than the sensitive variety due to consortium treatment compared to the irrigated condition. Pipecolate, nicotinate, l-methionine, and N-butylbenzenesulfonamide were accumulated significantly higher in the tolerant variety than in the sensitive variety under consortium treatment compared to drought.

**Table 5:** Fold Change results for the top 15 metabolites identified through partial least square discrepant analysis (PLS-DA), random forest (RF) and significant analysis of metabolites (SAM).

Metabolites	SAM (d-value)	PLS-DA VIP score (variance For component 1)	RF (Mean Decrease Accuracy)	Sensitive Variety (Pb Noor-2009)		Tolerant Variety (93127)	
				Consortium/Control	Consortium/Drought	Consortium/Control	Consortium/Drought
L-Histidine	13.699	0.78134	0.022891	0.6259	2.0353	0.95862	1.7102
Leu Pro	7.4422	1.2096	0.02324	0.45916	3.0445	1.7947	2.3216
Glycerol	4.5958	0.80756	0.0123	1.7003	0.46959	2.5975	0.55017
Citrulline	8.9761	0.561	0.0098248	1.3648	2.2151	0.73095	2.1002
Pipecolate	2.6301	0.17334	0.001843	0.80534	1.49718	0.87957	2.1193
Riboflavin	5.8685	0.58757	0.0053125	1.91284	0.033231	2.8154	0.2145
3-Hydroxy-3-Methylglutarate	11.611	0.53419	0.013604	1.6331	0.58684	2.1484	1.3843
L-Asparagine	0.58759	0.38126	0.00055552	1.6521	1.138	1.9976	0.55303
5-Oxo-L-Proline	1.1228	0.070754	0.00021442	0.76712	1.9273	1.52248	2.3664
Nicotinate	2.272	0.081542	0.0043686	0.76863	1.5513	0.89901	2.3142
Aspartate	8.6665	0.63323	0.027177	1.92148	0.31464	3.1182	1.22958
Nicotinamide	0.61979	0.042192	0.019595	1.6602	0.33112	1.4331	0.2246
Threonine	16.51	0.27547	0.042461	0.9468	1.68094	0.99299	1.9921
L-Methionine	8.7535	0.70495	0.019143	0.86428	2.3553	2.3026	5.2437
N-Butylbenzenesulfonamide	8.6504	2.0704	0.0066463	3.6937	0.926	2.1847	1.5272

The metabolites showing the greatest increase or decrease in metabolite concentration in the leaves of consortium plants compared to control and drought treated plants in two different varieties.

### Metabolic Pathway Analysis

To better elucidate the biological functions of identified metabolites, a pathway analysis was performed using *Arabidopsis thaliana* as the pathway library. Table 6 shows the metabolites involved in each pathway, number of hit metabolites, and FDR of the pathway. As expected, these metabolites were involved in a number of different pathways (Table 6). The pathway analysis using MetaboAnalyst was able to mine metabolites existing in seven biosynthesis pathways, namely Aminoacyl-tRNA biosynthesis, Isoquinoline alkaloid biosynthesis, Phenylalanine, tyrosine and tryptophan biosynthesis, Lysine biosynthesis, Glucosinolate biosynthesis, Indole alkaloid biosynthesis, Tropane, piperidine, and pyridine alkaloid biosynthesis. In addition, twelve primary metabolisms such as the glycine, serine, and threonine metabolism, Arginine and proline metabolism, Alanine, aspartate and glutamate metabolism, Nicotinate and nicotinamide metabolism, Cysteine and methionine metabolism, Tyrosine metabolism, Phenylalanine metabolism, Arginine and proline metabolism, Riboflavin metabolism, Cyanoamino acid metabolism, Beta-Alanine metabolism, Glycerolipid metabolism, and Nitrogen metabolism were also significantly altered due to drought and consortium treatments.

**Table 6:** Pathway names, total metabolites involved in that pathways, metabolites significantly accumulated in present study (hits), and false discord rate (FDR).

Pathway name	Total	Hits	FDR
Aminoacyl-tRNA biosynthesis	67	9	6.4825E-5
Glycine, serine and threonine metabolism	30	4	0.010825
Isoquinoline alkaloid biosynthesis	6	2	0.0109
Phenylalanine, tyrosine and tryptophan biosynthesis	21	3	0.02069
Alanine, aspartate and glutamate metabolism	22	3	0.02109
Lysine biosynthesis	10	2	0.028252
Nicotinate and nicotinamide metabolism	12	2	0.032452
Cysteine and methionine metabolism	34	3	0.03566
Tyrosine metabolism	18	2	0.037935
Carbon fixation in photosynthetic organisms	21	2	0.038368
Glucosinolate biosynthesis	54	3	0.041917
Indole alkaloid biosynthesis	7	1	0.043634
Tropane, piperidine and pyridine alkaloid biosynthesis	8	1	0.044634
Phenylalanine metabolism	8	1	0.05634

## Discussion

Drought affects physiological and biochemical processes in plants, thus resulting in altered growth and development. Our previous study demonstrated that the PGPR or PGRs could play a role in reducing drought effect, however, the synergetic effect of PGPR and PGRs was more effective in alleviating drought stress in chickpea^35^. Understanding the synergistic effects of growth promoting rhizobacteria along with plant growth regulating hormones on physiological and biochemical properties can play a vital role in improving drought tolerance in chickpeas. Our study is the first step towards such understanding.

Drought stress enhanced damage to chlorophyll and photosystem, and decreased root and shoot biomass accumulation. The consortium of PGPR and PGRs played a significant positive role and augmented the shoot dry weight to be much greater than the controls in both the varieties. The root biomass accumulation induced by consortium of PGPR and PGRs was same as irrigated control, but was significantly higher than drought control which indicates that the consortium treatment potentially helped plants to uptake water and nutrients from the soil, and thus maintained growth under stress conditions. PGPR induced increase in shoot and root weight has been previously demonstrated^35,45–51^. Our study demonstrated 75% increase in dry matter content when PGRs were applied in combination with PGPR. The combined effect of PGPR + PGRs improved the water budget of the plant (as demonstrated by RWC), which ultimately resulted in higher growth and increased dry biomass in both varieties compared to the untreated drought stressed plants. The sensitive variety responded more to the consortium than that of the tolerant variety. Though PGRs and PGPR treatment positively influenced sensitive genotype more than tolerant genotype; however, the tolerant genotype still showed higher values for chlorophyll content, chlorophyll fluorescence and RWC. The combined effect of PGPR and PGRs helped plants to maintain water balance, and intact photo- and root system, which ultimately resulted in higher growth under drought condition. Previously, researchers reported increased growth in plants due to PGPR or PGRs treatments when compared to drought stress^52–54^.

Environmental stresses significantly decrease leaf sugar content thereby causing physiological and biochemical alterations, as sugar preserves the structure of macromolecules and membranes during extreme dehydration^55^. Previous studies suggest that PGPR-accumulated soluble sugars led to drought tolerance in plants as the soluble sugars or sugar derivatives acted as an osmoprotectant under water stress conditions^56,57^. Silvente *et al*.^58^ attributed the depletion of sucrose in soybean leaves to the decrease in dry weight under drought conditions. A clear increase in leaf sugar content was evident by both varieties in our study treated with PGPR + PGRs. Soluble sugars do not only act as metabolic resources and structural constituents of cells but also function as signals, regulating many processes related with plant growth and development under water deficit condition^59^. An increase in sugar concentrations in the leaf also activates the expression of genes related to photosynthetic activities^60^. The role of Put in accumulation of sugar in plant leaves under stress condition has also been reported previously^61,62^. Khosravi *et al*.^63^ demonstrated that SA treatment disrupts the enzymatic system of polysaccharide hydrolysis and thus results in increased sugar levels which may lead to osmotic balance under stress conditions. The present study specifies the first ever report of the combined treatment of PGPR and PGRs on soluble sugar content. The PGPR and PGRs combination induced higher sugar accumulation which potentially acted as an osmoprotectant (demonstrated by RWC) in photosynthetic organs and helped maintain photosynthetic efficiency, resulting in increased growth under drought conditions.

The role of PGPR + PGRs is noteworthy in reducing the lipid peroxidation as measured by the malondialdehyde (MDA) content. Lipid peroxidation is an oxidative degradation process of lipids that results in the production of reactive oxygen species (ROS). The most common targets of lipid peroxidation are biological membranes. The peroxidation process significantly damages cell membrane structure and function. These modifications lead to functional changes that disrupt cellular metabolism. Singh & Jha *et al*.^64^ recorded an increase in lipid peroxidation in wheat with increases in salt concentration; however, inoculation with *Pseudomonas pseudoalcaligenes* and *Bacillus pumilus* showed significant effects to overcome salt stress by reducing the lipid peroxidation. Similarly, a combined treatment of SA and Put was more effective in reducing lipid peroxidation than SA or Put alone. Put reduced oxidative damages by reducing lipid peroxidation had been reported earlier by Tang *et al*.^65^. PGPR or PGRs treatment can reduce the lipid peroxidation by 33%, whereas co-inoculation of PGPR can reduce it by 50%^66,67^. Present investigation demonstrated 82% decrease in lipid peroxidation which suggests that a combined treatment of PGPR + PGRs is more effective in controlling oxidative damage to cells than PGPR or PGR used alone. The role of the combined effects of PGPR and PGRs in controlling oxidative damage in our study was further evidenced by the inhibition of the production of antioxidant enzymes (CAT, POD, SOD, and APOX). Antioxidant enzymes play critical role in the detoxification of the harmful effects of reactive oxygen species which are produced in response to environmental stresses. PGPR and PGRs consortium reduced the antioxidant enzyme activities in both tolerant and sensitive varieties. PGPR mediated decreases in antioxidant enzyme activity had been reported previously in other plants including canola, cucumis, wheat, and barley^68,69^, but their effect in combination with PGRs has not been studied. PGPR + PGRs treatment ameliorated drought induced decreases in protein content in both varieties in our study. The higher level of protein accumulation was evidenced to influence various physiological mechanisms such as, osmotic adjustment, ROS detoxification, and regulation of the intracellular pH level; thus, it is considered to enhance stress resilience in plants^41^. A positive correlation between increased protein levels and their involvement in ROS scavenging and oxidative stress metabolism was demonstrated by Ford *et al*.^70^. This ability of PGPR + PGRs may be attributed to the fact that PGPR + PGRs significantly reduces the production of ROS under stress which causes degradation of proteins, lipids, and nucleic acid^71^. PGPR + PGRs provides drought tolerance in our study by producing increased levels of protein content in photosynthetic tissues that reduced ROS damage to photosynthetic structure. This change led to an increase in the photosynthetic rate and cell viability, and increased growth and productivity under drought conditions.

In order to elucidate the comparative metabolic study to link plant function to metabolic status and to enhance our understanding of the effect of PGPR + PGRs consortium on chickpea metabolites grown under drought conditions, a metabolic profiling of the two chickpea varieties (i.e., drought sensitive and tolerant) was performed. The altered level of metabolites (increases or decreases) treated with PGPR + PGRs consortium under drought conditions could potentially be associated with the different biochemical pathways involved in controlling stress tolerance in chickpea. Modulation in amino acid of plants grown under stress conditions has been reported to be involved in different mechanisms, such as osmotic adjustment, detoxification of reactive oxygen species, and intracellular pH regulation^58,72,73^. Under stress conditions, amino acids are targets of oxidation in photosynthetic machinery that usually generates different ROS, such as singlet oxygen, superoxide, and peroxide. Drought stress can generate these ROS in such a high level that the D1 protein production is inhibited which is essential for damage repair in photosystem II^74,75^. The increased amino acid levels (threonine, methionine, histidine, etc) were accompanied by high levels of protein accumulation due to the PGPR and PGRs combined treatment in our study, which potentially contributed significantly to the ROS scavenging mechanism and maintained healthy photosynthetic machinery under drought stress conditions.

In the present study, L-asparagine increased in chickpea plants treated with PGPR + PGRs consortium. Asparagine can be transaminated, particularly in leaves, to yield oxosuccinamic acid, which may then be reduced to hydroxysuccinamic acid and subsequently deaminated to yield malate. Asparagine plays a vital role in tissues requiring nitrogen for growth such as developing seeds, roots, and leaves. It had been reported earlier that asparagine accumulation in legumes regulate N_2_-fixiation under drought conditions^76^. Asparagine also plays a critical role in nitrogen transport and in various physiological processes including growth initiation and nitrate assimilation in leguminous plants^77–79^. Higher accumulation of asparagine could be PGPR + PGRs-induced drought stress response strategy in plants. Our findings are in agreement with Forde & Lee^70^ and Curtis *et al*.^80^, who reported enhanced accumulation of asparagine in response to various biotic and abiotic stresses.

The increase in the vitamins riboflavin and nicotinamide in PGPR + PGRs treated plants was significant under drought conditions. These metabolites have lower concentrations in untreated drought stressed plants. Riboflavin is required for normal plant growth and development. Riboflavin accumulation reduces the levels of ROS, lipid peroxides and increases sugar content, thereby enhancing drought tolerance in plants^81^. Guhr *et al*.^82^ noted significant increases in the drought tolerance of *Agaricus bisporus* when treated with riboflavin. This effect was even stronger in the combined treatment of PGPR + PGRs. Nicotinamide is a stress-associated compound that can induce and regulate secondary metabolic accumulation^83^. Farhat *et al*.^84^, demonstrated that nicotinamide induced drought tolerance, protected the photosynthetic pigments, and increased the metabolic activities relevant to growth under stressed conditions. Nicotinamide is responsible for increases in total soluble carbohydrates and proteins and has a positive correlation with increases in leaf and root area^85^. It acts as a stress signal and plays a defensive role in plants because of its involvement in many enzymatic oxidation-reduction reactions in plants. Increased levels of nicotinamide promotes a decrease in cellular levels of H_2_O_2_^86^. The increased levels of nicotinamide and riboflavin in our study, due to combined PGPR and PGRs treatment, potentially contributed to reduced lipid peroxidation and ROS levels, and increased photosynthesis that helped in maintaining growth under drought stress conditions.

A decreased level of sugar (glucosamine) was reported in untreated plants grown under drought stress conditions. The reduced level of common sugar accumulation was further accompanied by reduced concentrations of the organic acids (i.e., tartaric acid and citric acid) and some amino acids (tyrosine, methionine, histidine) in leaves of untreated stressed chickpea plants. Sugar depletion normally occurs during environmental stresses that lead to a significant decrease in the efficiency of photosynthesis in source tissues, thus reducing the supply of soluble sugars in sink tissues. Under conditions of sugar deprivation, substantial physiological and biochemical changes occur to sustain respiration and other metabolic processes^58,87^. Both drought sensitive and tolerant varieties accumulated relatively high amount of sugar when treated with PGPR + PGRs consortium, enabling plants to withstand harsh environmental conditions. This has also been evident from our leaf sugar content estimation, where significant accumulation of sugar was noted in PGPR + PGRs treated plants. Sugar acts as a signalling molecule and, in plants, different sugar signals are generated by carbon metabolism and photosynthesis in source and sink tissues to modulate growth, development, and responses of the plant to various stresses^88^. Sugar also acts as an osmoprotectant under water stress conditions^57^. Drought stress affected photosynthetic capacity (demonstrated by reduced chlorophyll fluorescence and chlorophyll content) and water balance (demonstrated by reduced RWC) in both varieties; however, the level of reduction was greater in the sensitive variety than in the tolerant variety. The PGPR + PGRs treatment produced significantly higher sugar content than the drought and irrigated control in both varieties. The increase in sugar content helped to maintain a healthy photosynthetic system and water balance in chickpea plants in our study, demonstrating a significantly increased growth rate compared to the drought stress condition treatment.

In the present study, the PGPR and PGRs treatment enhanced the accumulation of N-acetyl putrescence in the sensitive variety compared to untreated plants. The role of Put in plant growth, development, and responses to abiotic stresses has been documented in plants^89,90^. Drought tolerant variety exhibited high accumulations of pipecolate when exposed to drought stress. The increases in pipecolate in water-stressed leaves of the tolerant variety is vital as it has a defensive action on proteins and nucleic acid structures by maintaining a stable osmotic status in plants under variable soil water and salt stresses^91,92^. Higher concentrations of pipecolate in plants regulates inducible plant immunity and acts as an indicator of abnormal protein metabolism in diseased plants^93,94^. Rahman *et al*.^40^ found significantly altered levels of pipecolate in the leaves of wheat grown under water stress conditions.

Biosynthesis and metabolic pathways of aromatic amino acids such as phenylalanine, tyrosine, and tryptophan; alanine, aspartate, and glutamate were upregulated in PGPR and PGRs treated plants. It is a high flux bearing pathway and has been estimated that more than 30% of all fixed carbon is directed through this pathway^95^. The glycine, serine, and threonine pathway was downregulated in the present study. This pathway plays a key role in the synthesis of amino acids including lysine, threonine, methionine, and isoleucine^96^. Nicotinate and nicotinamide metabolism was upregulated in the present study. Nicotinate and nicotinamide are essential for organisms as they are precursors for the generation of coenzymes, NAD^+^ and NADP^+^. These coenzymes are crucial for many metabolic pathways including glycolysis, TCA cycle, pentose phosphate cycle, fatty acid biosynthesis, and metabolism pathways and many others^97^. Phenylalanine metabolism is of great importance for plants under drought stress condition. Phenylalanine is used as a protein building block but it is also a precursor for numerous plant compounds that are crucial for plant reproduction, growth, development, and defence against different types of stresses^98,99^. The levels of leucyl proline, citrulline, and threonine were amplified in chickpea plant leaves under stress conditions and organic acid had a negative correlation with water scarcity. Integrative use of active PGPR strains and PGRs seems to be a promising eco-friendly strategy for increasing growth and drought tolerance in crop plants grown in sandy soil.

In conclusion, our data evidenced that both tolerant and sensitive genotypes were benefitted by the synergistic effect of PGPR and PGRs, and demonstrated increased shoot and root growth. The synergistic effect of PGPR and PGRs also ameliorated drought effect by reducing the degradation of chlorophyll content and lipid peroxidation, by improving water balance and osmoregulation, and by increasing production of protein which can effectively reduce damaging effect of ROS and can help in maintaining healthy photosystem. The non-targeted UPLC–HRMS global metabolomic profiling and multivariate analysis identified metabolites those altered due to the synergistic effect of PGPR and PGRs, and potentially improved drought tolerance in both varieties. Though there were genetic differences in altered level (>1.5 fold) of metabolites, the combined treatment of PGPR and PGRs significantly increased accumulation of histidine, proline, citrulline, nicotinate, threonine and methionine in both varieties compared to drought control. As these metabolites demonstrated enhanced level in both varieties due to the combined effect of PGPR and PGRs under drought conditions, they could potentially be associated with drought tolerance in chickpea. Pipecolate only showed significant accumulation in tolerant variety, not in sensitive variety, which could be due to the inherent genetic difference between two varieties. Contrary to the observation of comparing drought vs consortium treatment, proline, glycerol, riboflavin, 3-Hydroxy-3-Methylglutarate, aspergine, aspartate, methionine and N-Butylbenzenesulfonamide showed higher accumulation in both varieties due to consortium treatment compare to irrigated control. The altered level of these metabolites could potentially increase the performance of both varieties treated with consortium and is demonstrated by producing higher shoot biomass, protein and sugar content compare to irrigated control. Our results demonstrated that the enhanced accumulations of different metabolites due to synergistic effect of PGPR and PGRs, could potentially be associated with increased root and shoot biomass production, enhanced osmoregulation and RWC, increased protein and sugar production, and provide intact photosystem in both varieties. Although some of these identified metabolites are promising as biomarkers for improving drought tolerance in chickpea, their correlation to drought tolerance in chickpea and other pulses requires further investigation. These data provide information that may, with further investigation, help to understand the biochemical pathway underlying stress tolerance in chickpea.

## Materials and Methods

The experiment was performed during the 2015–2016 chickpea growing seasons. Seeds of two chickpea varieties (Punjab Noor-2009 and 93127) differing in sensitivity to drought were obtained from Ayub Agriculture Research Institute, Faisalabad. Bacterial colonies were isolated from the rhizosphere of chickpea plants grown in sandy soil of Karak, Bhakkar, and Cholistan (with 7%, 6%, and 4% soil moisture contents) respectively, and were named as P1 (*Bacillus subtilis*), P2 (*Bacillus thuringiensis*), and P3 (*Bacillus megaterium*). The experiment was carried out in a Randomized Complete Block Design (RCBD) with a plot size of 5 × 1.5 m^2^ with six replicates and three treatments (Table 7).

**Table 7:** Experimental work plan.

Control	Plants grown under normal condition (irrigated)
Consortium	Plants grown under drought stress and treated with consortium of 3 PGPR (*Bacillus subtilis*, *Bacillus thuringiensis*, and *Bacillus megaterium* and 2 PGRs (salicylic acid and putrescine)
Drought	Untreated uninoculated plants grown under drought stress

### Collection of Soil Samples

Soil samples were collected at 6 inches from top soil from three rain-fed areas (Karak, Bhakkar, and Cholistan) of Pakistan with 7%, 6%, and 4% of soil moisture contents, respectively^36^.

### Isolation and Growth of Bacteria from Soil

Serial dilution method was followed for isolation of bacteria from three different soil samples. The soil sample (1 g) was suspended in distilled water (9 mL), stirred for 1 h with a magnetic stirrer, and the soil suspension thus obtained was centrifuged (3000 rpm) for 10 min. Decimal dilutions were prepared from the supernatant and aliquots (20 μL) were spread on Luria-Bertani (LB) agar plates and incubated for 2 days. Colonies that appeared on LB agar plates were streaked 4–5 times till a single pure colony appeared.

### Sterilization of Seeds

Seeds were surface sterilized with 70% ethanol, followed by soaking in 10% chlorox for 2–3 mins and washed several times with autoclaved distilled water.

### Method of Inoculation

The Luria Bertani (LB) broth was inoculated with fresh (24 h old) bacterial culture. The three isolated bacterial strains were used in equal amount. The inoculated LB broth incubated in the shaker for 48 h at 27 °C followed by centrifugation at 10,000 rpm (10 min). Pellet was mixed with distilled water and the OD (at 660 nm) was adjusted to 1. The colony was then soaked in broth with isolates. The seeds were soaked in broth for 3 h prior to sowing.

### Characterization of Bacterial Isolates for Beneficial Plant Growth Promoting Traits

#### Colony Morphology and Gram staining of Isolated PGPR

Isolates were identified based on colony morphology and Gram staining. Picovskaya’s media was used for overnight growth of bacterial isolates and the isolates were placed on agar plates^100^. The color and shape of the colonies were recorded after 24 hours and the isolates were also checked for gram staining.

#### Catalase and Oxidase Test

The catalase and oxidase test was performed following the method of McFadden^101^ and Steel^102^.

#### IAA Production by Selected PGPR Strains

Indole-3-acetic acid (IAA) production by selected PGPR was determined by a colorimetric method using Salkowski’s reagent^103^.

#### Hydrogen Cyanide (HCN) Production by Selected PGPR Strains

Selected strains were screened for hydrogen cyanide production following the method of Lorck^104^.

#### Ammonia (NH3) Production by Selected PGPR Strains

The method of Cappuccino and Sherman^105^ was adopted for NH_3_ production.

#### Extraction, Purification and Characterization of Exopolysaccharides (EPS)

Mineral salt medium was used to culture the isolated bacteria^106^. The culture was then centrifuged at 15,000 rpm (10 min) after 10 days incubation period. The supernatant was mixed with 2-fold ice cold ethanol (95%) for EPS extraction and the complete precipitated solution was cooled at 4 °C and EPS was obtained from the above solution^107^. The extracted EPS were lyophilized with Labonco lyophilizer at 3000 psi and stored at room temperature^106^. Small quantities of lyophilized EPS were deferred in 2 mL of benzene, water, chloroform, acetone, ethanol, and methanol for determination of its solubility. The mixture was vortexed and stabilized for some time until the pellet development was observed.

#### Phosphate Solubilisation Index (PSI)

Pin point inoculation in the 24 h old culture of PGPR was made on Pikovskaya’s media and was transferred into sterilized petri plates. The inoculated plates were incubated for 7 days (28 °C). SI (solubilization index) was calculated by the formula of Pikovskaya^100^.

SI = colony diameter (cm) + halozone diameter (cm)/ colony diameter (cm).

#### Antibacterial Activity of Selected PGPR Strains

Agar well diffusion method^108^ was used for the determination of antibacterial activity of isolated PGPR.

#### Antifungal Activity of Selected PGPR Strains

For determination of antifungal activity of PGPR the agar tube dilution method was used^109^. The fungal strains used in this study were included:

1. *Helminthosporium sativum*
2. *Fusarium solani*

The fungal strains were maintained on Sabouraud Dextrose Agar (SDA) medium at 4 °C. The autoclaved test tubes were cooled (50 °C) and SDA was loaded with 67 μl of cell free supernatant pipette from the stock solution before solidification. The tubes were placed in slanting position and permitted to solidify at room temperature. One slant of the extract sample was prepared for each fungus species. The tubes with test compound and solidified media were inoculated with 4 mm diameter piece of inoculum, taken from a 7 days old culture of fungus. One sample of each extract was prepared, which were used for positive control. Slants with no extract were used as negative control. The test tubes were incubated at 28 °C for 7 days. The incubated test tubes were checked twice/week during the incubation period. Readings were taken by measuring the linear length of fungus in slant by measuring growth and growth inhibition was calculated with reference to negative control. Percent inhibition of fungal growth for each concentration was determined by the following formula:

Percent inhibition of fungal growth = 100 − Linear growth in test (mm)/Linear growth in control (mm) × 100.

#### Extraction of Bacterial DNA

A single colony of bacterial culture was used to inoculate tryptone yeast extract (TY) broth. The inoculated TY broth was incubated overnight in a shaker (Model: Excella E-24 incubated shaker). The grown culture was centrifuged at 12,000 rpm at 4 °C for (10 min) followed by suspending in lysis buffer. A 60 mL of NaCl (5 M) was added to the suspension and centrifugation was done again at 12,000 rpm for 10 min. The supernatant was transferred into a new tube followed by the addition of chloroform. The tube was inverted (50×) to mix well. The centrifugation was done twice by adding with 100% ethanol to clean the obtained DNA. The DNA was dissolved in distilled water. The purity of DNA was assessed through nanodrop spectrophotometry (260–280 nm)^110^.

#### PCR-amplification and 16S rRNA Sequence Analysis

Amplification of genomic DNA of bacterial isolates was done by the method of Weisburg *et al*.^111^. The primer used for PCR-amplification has the nucleotide sequence as fd1 (AGAGTTTGATCCTGGCTCAG) and rd1 (AAGGAGGTGATCCAGCC)^35^. The reaction mixture contained genomic DNA (50 μg), MgCl_2_ (1.5 mM), buffer (10×), Taq DNA polymerase (1 μ), dNTP mix (0.2 mM), and 10 moles of each primer. The volume was adjusted to 25 μL using autoclaved distilled water. The operating conditions were as follows: The 30 cycles of the reactions were repeated through the following reaction sequences: denaturation for 30 s at 94 °C, annealing at 55 °C for 30 s, extension for 2 min at 72 °C, and one additional cycle for chain elongation for 10 min at 72 °C. The amplified PCR products were electrophoresed on 1.2% (w/v) agarose gel with DNA ladder (1 kb) as molecular marker. The gel was stained with 0.01 gm/mL ethidium bromide and examined under UV trans-illuminator lamp.

#### Sequencing

Approximately 1400 bp purified PCR products were sequenced by using primers 27FAgAgTTTgATCMTGGCTCAg, 1492RTACggYTACCTTgTTACgACTT, 518FCCA gCAgCCgCggTA ATA Cg, and 800R TAC CAgggT ATC TAA TCC. Sequencing was accomplished by means of the Big Dye terminator cycle sequencing kit v.3.1 (Applied BioSystems, USA). Sequencing products were resolute on an Applied Biosystems model 3730XL automated DNA sequencing system (Applied BioSystems, USA) at the Macrogen, Inc., Seoul, South Korea.

#### Leaf Chlorophyll Content and Chlorophyll Fluorescence

SPAD chlorophyll meter (Spad-502 plus. Serial No. 20001472 made by Konica Minolta, Japan) was used for the determination of chlorophyll content in plant leaves. Chlorophyll fluorescence was measured on intact leaves of the abaxial surface (third leaf) after 30 min of dark adaptation with a pulse modular fluorometer (Model OS5‐FL, Opti‐ Sciences, Hudson, NH) in control, consortium and drought‐stressed leaves. Chlorophyll fluorescence and chlorophyll content were measured after 14 and 25 days of drought stress imposition at three leaflets in each plant and five plants per pot (a total of 15 readings) and averaged. The average value of 15 readings was considered as a single replication, and six replicated values/variety were used for statistical analysis and comparison of treatment means, and significant testing at *P < *0.05 level^21^.

#### Relative Water Content (RWC)

The relative water content (RWC) of leaves for each treatment was calculated according to the formula of Weatherly^112^.

RWC = [(fresh weight of leaves − dry weight of leaves)/(Turgid weight of leaves − dry weight of leaves)] × 100.

#### Measures of Shoot and Root Dry Weights

Shoots of five plants per replication were cut at the base and dried at 60 °C for 72 hr, and dry weight was taken by using an electronic scale. The roots of the same plants with soil were isolated, washed carefully to separate roots, dried at 60 °C for 72 hr, and weighed using an electronic scale. The root and shoot dry weights were measured after 25 days of drought stress imposition^21^.

#### Lipid Peroxidation (estimated as malondialdehyde)

The level of lipid peroxidation was determined by calculating the amount of malondialdehyde (MDA) formed by thiobarbituric acid (TBA) reaction as defined by Li^113^.

#### Extraction for Antioxidant Enzymes

For extraction of antioxidant enzymes, 0.5 g of leaves was grinded in 5 mL of 50 mM phosphate buffer by placing it on ice bath. The homogenate thus obtained was centrifuged at 4 °C for 20 min at 13000 g. The supernatant obtained was used for the determination of catalase, POD, and APOX activities.

#### Superoxide Dismutase Activity (SOD)

Superoxide dismutase (SOD) activity was determined following the method of Beauchamp & Fridovich^114^. Phosphate buffer (pH 7) containing 1% PVP was used to homogenize all the treated and control samples individually in four biological replicates. The homogenate was centrifuged at 4 °C for 15 min at 3,000 rpm and the supernatant was used for superoxide dismutase measurement at 560 nm after 20 min exposure in light, while the reference was treated in dark conditions. Absorbance was calculated by using the Nanodrop 1000 spectrophotometer.

#### Estimation of Leaf Protein, Proline, and Sugar Contents

The Bates *et al*.^115^ method was used for the estimation of proline content in leaves of chickpea, while the estimation of protein content was carried out following Lowery *et al*.^116^. Sugar content was estimated following the method of Dubois *et al*.^117^.

#### Leaf Tissue Collection and Sample Preparation for Metabolites

Leaf samples from control, consortium, and drought stressed plants were collected at midday for metabolic profiling. Leaves were harvested at 44 days (time point 1) and 60 days (time point 2) after the seed emergence. Sampled leaf tissues were frozen in liquid nitrogen immediately after collection and stored at −80 °C. Tissue samples were lyophilized for 72 hours and ground using a TissueLyser. Lyophilized powder (30 mg) was used for ultrahigh performance liquid chromatography-high resolution mass spectrometry (UPLC-HRMS) based metabolite profiling following the protocol of Lisec *et al*.^118^.

In brief, freeze dried leaf tissues were weighed (30 mg) into a clean Eppendorf tube, followed by the addition of internal standards (20 μL) to each sample. Methanol (750 μL) and ammonium acetate (10 mM, 750 μL) was added to each sample and vortexed for 1 min at room temperature. Centrifugation (17000 G, 10 m) was done after all the samples were ultra-sonicated for 20 min at room temperature. Supernatant (>1 mL) was transferred to a 1.5 mL Eppendorf tube, followed by a 50 μL transfer of supernatant to an Eppendorf tube. The supernatant was dried down after adding 50 μL of injection of standard solution. Samples were then vortexed (30 s) and put at 4 °C for 10 min, centrifuged at 20,000 rpm for 10 min, and the supernatant was transferred into a LC-vial.

#### UPLC–HRMS Analysis

Untargeted metabolomics profiling was performed on an ultrahigh performance liquid chromatography-high resolution mass spectrometry (Model: Thermo Ultimate 3000 UPLC and Thermo QExactive mass spectrometer) platform at the University of Florida Southeast Center for Integrated Metabolomics (SECIM). All samples were analyzed in positive and negative heated electrospray ionization with a mass resolution of 70,000 at *m/z* 200 as separate injections. Chromatographic separation was attained on an ACE Excel 2 C18 PFP100 × 2.1 mm, A 2 µm particle size column with mobile phase A as 0.1% formic acid in water, and mobile phase B as acetonitrile, at a flow rate of 350 µL/min with a run time of 16.8 min, mass resolution of 35,000 @ *m/z* 200, and mass range of 70–1000 *m/z*. Injection volume was 4 µL for negative ion mode and 2 µL for positive ion mode. The total run time per sample was 20.5 minutes. Probe (HESI probe) temperature was maintained at 350 °C for both positive and negative run with spray voltage of 3500 V and capillary temperature of 320 °C^21^.

#### Metabolite Data Analysis

Data tables with metabolite peaks (*mz*/*rt*) at 2 time points under control, consortium, and drought stress treatment were formatted as comma separated values (.csv) files and uploaded to the MetaboAnalyst 3.0 server (http://www.metaboanalyst.ca)^119^. To shrink any possible variance and to improve the performance for downstream statistical analysis, metabolite data generated by UPLC-HRMS were checked for data integrity and normalized using MetaboAnalyst’s normalization protocols (selecting normalization by sum, log transformation, and auto-scaling) for statistical analysis.

Univariate analysis (t-test and one way ANOVA) was applied to calculate the statistical significance of the metabolites between two groups means (consortium/control and consortium/drought). As the multivariate methods take all the variables into consideration, therefore, this method was applied for the comprehensive data analysis, for example, supervised methods-Partial Least Squares Discriminant Analysis (PLS-DA), Random Forest (RF) classification, and unsupervised method-Hierarchical clustering with heat map. A heat map was generated based on the Pearson distance measure and the Ward clustering algorithm, showing top 25 metabolites for consortium versus control and consortium versus drought treatments by PLS-DA VIP (variable importance in projection) score using a significance level of *P* ≤ 0.05, and post-hoc analysis of Fisher’s LSD. The samples were arranged according to their sampling time points (time point 1 and 2) in all two groups. The important metabolites were identified by using 3 different methods separately: SAM (Significant Analysis of Metabolites), PLS-DA, and RF.

The pathway analysis was performed using MetaboAnalyst for the identified important metabolites using *Arabidopsis thaliana* pathway libraries. The Kyoto Encyclopedia of Genes and Genomes (KEGG) pathway database (http://www.genome.ad.jp/kegg/pathway.html) was also used for the metabolites that were not found in the *Arabidopsis* pathway libraries.

#### Data Analysis for Biochemical Characters

Experiments were repeated six times. The data analysis was carried out by using software Statistics, version. 8.1. An ANOVA was performed to determine the effect of treatments and error associated with the experiment. To identify significant differences among treatments, a mean comparison of traits was carried out by using protected LSD (*p* = 0.05) test where the error mean square was used to estimate the standard error of differences between mean.

## Supplementary information

S1
