# Supplementary material for: Comparative Physiological and Metabolic Analysis Reveals a Complex Mechanism Involved in Drought Tolerance in Chickpea (Cicer arietinum L.) Induced by PGPR and PGRs
Source: Sci Rep. 2019 Feb 14;9:2097. doi: 10.1038/s41598-019-38702-8 (PMC6376124; doi:10.1038/s41598-019-38702-8)
Supplement: Supplementary file 1 — S1 [file 41598_2019_38702_MOESM1_ESM.pdf]

Comparative Physiological and Metabolic Analysis Reveals a Complex Mechanism Involved in Drought Tolerance in Chickpea (*Cicer arietinum L.*) Induced by PGPR and PGRs

Running Title: Physiological and Metabolic Changes Induced by PGPR and PGRs in Chickpea

Naeem Khan[1], Asghari Bano [2*], M Atikur Rahman[3], Jia Guo[3], Zhiyu Kang[4] and Md Ali Babar[3*]

[1]Department of Plant Sciences, Quaid-I-Azam University, Islamabad, Pakistan

[2]Department of Biosciences, University of Wah, Wah Cantt, Pakistan

[3]Department of Agronomy, IFAS, University of Florida, Gainesville, FL, USA

[4]Zhiyu Kang, College of Agronomy and Biotechnology, Yunnan Agricultural University,

Kunming, Yunnan province (650201), China

Corresponding authors: *mababar@ufl.edu and *banoasghari@gmail.com

S1. List of 55 significantly different levels of metabolites (ANOVA, P ≤ 0.05) with their molecular formula, compound type, p-value, false discovery rate (FDR) and fold change (consortium/control and consortium/drought) in chickpea leaves under control, consortium and drought conditions.

S#	Metabolites	Compound type	Molecular Formula	SENSITIVE VARIETY (PB-NOOR-2008)						TOLERANT VARIETY (93127)					
				p.value	FDR	Fold Chang (Consortium/Control)	p-value	FDR	Fold Change (Consortium/Drought)	p.value	FDR	Fold Change (Consortium/Control)	p-value	FDR	Fold Change (Consortium/Drought)
1	Threonine/Homoserine	AMINO ACID	C4H9NO3	0.042454	0.045353	0.9468	1.64E-05	0.00014141	1.68094	1.67E-07	0.049582	0.99299	1.64E-05	0.00014141	1.9921
2	L-Asparagine	AMINO ACID	C4H8N203	0.001829	0.04471	1.6521	0.03234	0.04037	1.138	0.00018936	0.0029541	1.9976	0.044253	0.01443	0.55303
3	BOC-D-	AMINO	C14H19	0.002	0.002	0.86839	0.000	0.002	1.1214	0.0382	0.00299	1.7882	0.00815		1.96116

	Phenylalanine	ACID	NO4	2534	854		876	732		49			46	0.03742	
4	Alanine/Sarcosine	AMINO ACID	C3H7NO2	0.042483	0.010348	1.5029	1.81E-05	0.00014141	0.60541	0.00079207	0.0077227	1.82054	1.81E-05	0.00014141	0.6043
5	BOC-L-Tyrosine	AMINO ACID	C14H19NO5	0.00334	0.029835	1.0076	0.00108	0.0032084	1.4214	NS	NS	NS	0.00108	0.0032084	0.75756
6	L-Methionine	AMINO ACID	C5H11NO2S	0.022453	0.032457	0.86428	4.05E-08	6.42E-07	2.3553	4.18E-06	0.00010869	2.3026	4.05E-08	6.42E-07	5.2437
7	Aspartate	AMINO ACID	C4H7NO4	0.025845	0.004748	1.92148	2.67E-07	3.47E-06	0.31464	0.00040921	0.0053197	3.1182	2.67E-07	3.47E-06	1.22958
**8**	L-Histidine	AMINO ACID	C6H9N3O2	0.012467	0.045097	0.6259	5.70E-05	0.00034201	2.0353	0.0014049	0.027589	0.95862	5.70E-05	0.00034201	1.7102
9	5-OXO-L-Proline	AMINO ACID	C5H7NO3	0.0039265	0.028199	0.76712	0.0021348	0.0055505	1.9273	0.011939	0.0055505	1.52248	0.0021348	0.0055505	2.3664
10	Tryptophan-2,3,3-D3	AMINO ACID	C11H12N2O2	4.87E-07	3.85E-05	0.038543	0.005072	0.011635	4.91136	1.52E-08	0.027589	2.811	0.0012404	0.0034555	7.2263
11	Proline	AMINO ACID	C5H9NO2	0.0016791	0.020758	0.53456	0.033542	0.029574	4.5811	0.0031431	0.027589	1.15437	0.053542	0.03177	2.2191
12	L-Leucine 13C6	AMINO ACID	C6H13NO2	NS	NS	NS	0.00025037	0.0011488	1.2866	NS	NS	NS	0.00025037	0.0011488	1.0392
13	L-Isoleucine	AMINO ACID	C6H13NO2	0.003143	0.022875	1.10047	0.020013 0.0011488	0.0093853	1.9031	0.033942	0.023862	0.774	0.003971	0.009385	1.8007

14	4-Acetamidobutanoate	AMINO ACID	C6H10NO3	0.0418593	0.048256	0.9515	0.032844	0.042534	1.7819	0.038544	0.042954	1.0789	0.02524	0.04262	1.7314
15	5-Aminolevulinic Acid	NON PROTIEN AMINO ACID	C5H9NO3	0.03325	0.027698	1.3586	0.022144	0.018648	1.2418	0.024195	0.041995	1.1983	0.026456	0.03456	0.48264
16	Nicotinate	ORGANIC COMPOUND	C6H5NO2	0.029868	0.035132	0.76863	0.00074612	0.0025303	1.5513	0.012431	0.03265	0.89901	0.00074612	0.0025303	2.3142
17	4-Hydroxy-L-Phenylglycine	CHEMICAL COMPOUND	C8H9NO3	0.018467	0.009573	1.8117	3.55E-05	0.00025173	0.4991	0.0028267	0.031001	1.2659	3.55E-05	0.00025173	0.99374
18	L--Lactic Acid	ORGANIC COMPOUND	C3H603	0.010375	0.045097	0.61998	0.00715	0.014802	1.8606	NS	NS	NS	0.00715	0.014802	1.3207
19	Phenylpyruvate	ORGANIC COMPOUND	C9H8O3	0.015042	0.049512	0.76334	0.0034132	0.0085882	1.3726	0.040726	0.019322	0.8137	0.0034132	0.0085882	1.0965
20	Dibutyl-phthalate	ORGANIC COMPOUND	C16H22O4	NS	NS	NS	0.0018551	0.0049895	1.2062	0.035493	0.049524	1.0197	0.0018551	0.0049895	1.0148
21	Syringic Acid	CHEMICAL COMPOUND	C9H10O5	NS	NS	NS	0.04608	0.041471	1.3422	0.041255	0.036265	0.74377	NS	NS	NS
22	Allantoin	CHEMICAL	C4H6N4O3	1.92E-05	0.0006913	**0**.83083	0.020	0.042	1.771	NS	NS	NS	0.00620		2.3143

		COMPOUND			3		082	634					82	0.004922	
23	2-Aminophenol	ORGANIC COMPOUND	C6H7NO	0.022424	0.043953	1.0109	NS	NS	NS	0.0093575	0.056145	0.97942	0.041363	0.02634	1.4987
24	Glutarate	ORGANIC COMPOUND	C5H8O4	0.032423	0.035246	0.96281	0.019132	0.029261	**1.9742**	0.04922	0.0.36224	1.3571	0.019132	0.029261	1.8798
25	Citrulline	ORGANIC COMPOUND	C6H13N3O3	0.037474	0.015942	1.3648	1.06E-06	1.18E-05	2.2151	NS	NS	NS	1.06E-06	1.18E-05	2.1002
26	3,5-Di-tert-butylbenzaldehyde	CHEMICAL COMPOUND	C15H22O	NS	NS	NS	0.0085414	0.01606	0.55734	0.0082513	0.02342	2.836	0.0085414	0.01606	1.9226
27	D-SacchariC Acid	CHEMICAL COMPOUND	C6H10O8	0.0026276	0.020758	0.75914	0.018537	0.029062	1.2337	NS	NS	NS	0.018537	0.029062	0.89319
28	Triethyl phosphate	CHEMICAL COMPOUND	C6H15O4P	0.038213	0.012749	0.92564	0.0077088	0.015418	1.3068	NS	NS	NS	0.0077088	0.015418	1.0519
29	N-Butylbenzenesulfonamide	CHEMICAL COMPOUND	C10H15NO2S	0.012987	0.045097	**3.6937**	0.029854 0.13047	0.02433	0.926	0.0014376	0.027589	2.1847	0.013047	0.16153	1.5272
30	Pipecolate/L-Pipecolic Acid	ORGANIC MOLECULE	C6H11NO2	0.02537	0.003951	0.80534	0.043969	0.0163	1.73412	0.001507	0.013061	0.87957	0.013969	0.0045325	2.1193

31	Dopamine	ORGANIC CHEMICAL	$C_8H_{11}NO_2$	0.004254	0.029875	1.47561	0.04958	0.02424	0.04532	0.012345	0.03801	1.1155	0.005323	0.037442	0.7977
32	Tartaric Acid	ORGANIC ACID	$C_4H_6O_6$	0.023958	0.048373	1.2327	NS	NS	NS	0.041879	0.02744	0.825	0.032789	0.049223	1.0949
33	Citrate	ORGANIC ACID	$C_6H_8O_7$	0.034865	0.046286	0.78882	NS	NS	NS	0.014744	0.00642	1.9457	0.0072112	0.014802	1.5867
34	Cytidine	NUCLEOSIDE	$C_9H_{13}N_3O_5$	0.0025008	0.020758	0.55976	0.032535	0.04745	0.99644	0.026247	0.03993	0.59427	0.049875	0.048785	1.0161
35	Adenosine	NECLEOSIDE	$C_{10}H_{13}N_5O_4$	NS	NS	NS	0.0079924	0.015585	1.4215	0.02768	0,044523	0.58496	0.0079924	0.015585	0.91276
36	Guanine	NUCLEOBASE	$C_5H_5N_5O$	0.013129	0.045097	1.8827	0.006474	0.014027	2.2203	NS	NS	NS	0.0064741	0.014027	1.0069
37	Succinate	DICARBOXALIC ACID	$C_4H_6O_4$	0.023693	0.01543	0.62089	0.012398	0.020575	2.2754	0.03437	0.033431	0.57205	0.012398	0.020575	1.2616
38	3-Hydroxy-3-Methylglutarate	DICARBOXALIC ACID	$C_6H_{10}O_5$	0.011511	0.017476	1.6331	3.49E-09	1.20E-07	0.58684	0.02971	0.005242	2.1484	3.49E-09	1.20E-07	1.3843
39	Glyceric Acid	SUGAR ACID	$C_3H_6O_4$	0.02426	0.041253	0.88825	0.0036596	0.0089202	1.6064	NS	NS	NS	0.0036596	0.0089202	1.6045
40	Glucosamine/Mannosamine	AMINO SUGAR	$C_6H_{13}NO_5$	0.01735	0.041873	2.97119	0.00024217	0.0011488	1.2702	0.031245	0.025252	3.5942	0.00024217	0.0011488	2.1578

41	Nicotinamide	VITAMIN	C6H6N2O	0.005424	0.011293	1.6602	4.12E-08	6.42E-07	0.33112	0.036834	0.095253	1.4331	4.12E-08	6.42E-07	0.2246
42	Riboflavin	VITAMIN	C17H20N406	0.011032	0.011943	1.91284	4.63E-09	1.20E-07	0.033231	0.007823	0.035477	2.8154	4.63E-09	1.20E-07	0.2145
43	Ascorbic Acid	VITAMIN	C6H8O6	0.022624	0.003774	1.05779	3.98E-06	3.88E-05	1.51905	0.014736	0.044238	0.41135	3.98E-06	3.88E-05	0.45511
44	4-Coumarate	ENZYME	C18H16O4	NS	NS	NS	0.038573	0.0025834	1.195	NS	NS	NS	0.044321	0.015475	1.1891
45	Alpha-Ketoglutaric Acid	DERIVATIVE	C5H6O5	0.043861	0.11244	1.2488	NS	NS	NS	0.02418	0.025751	0.54993	0.036332	0.031242	1.0591
46	N-Methyl-L-Glutamate	DERIVATIVE	C6H11NO4	0.0064138	0.038976	3.6462	0.0061121	0.013621	0.53655	0.0044003	0.034763	2.124	0.0061121	0.013621	0.65104
47	Erucamide	FATTY ACID DERIVATIVE	C22H43NO	NS	NS	NS	0.04736	0.04334	1.2266	NS	NS	NS	0.02736	0.03955	0.96369
48	Isocytosine	PYRIMIDINE BASE	C4H5N3O	0.0022901	0.020758	0.55904	0.043635	0.023467	1.0098	NS	NS	NS	0.043564	0.049852	1.0193
49	Uridine	PYRAMIDINE ANALOG	C9H12N2O6	0.0086861	0.045097	0.61422	0.0094668	0.017172	1.4615	0.044183	0.032573-	0.46259	0.0094668	0.017172	1.1186
50	Isocitric Acid	ISOMER	C6H8O7	NS	NS	NS	0.011934	0.020237	1.7403	0.03964	0.043734	0.82764	0.011934	0.020237	1.0791
51	Glycerol	POLYOL	C3H8O3	0.00074328	0.011744	1.7003	0.0002719	0.0011784	0.46959	0.0067165	0.047626	2.5975	0.021339	0.032008	0.55017

							4								
52	N-Acetylputrescine	POLYAMINE	C6H14N2O	0.0002211	0.0043668	0.75027	0.00073549	0.0025303	1.4475	NS	NS	NS	0.010852	0.01881	1.6451
53	Leu Pro	PEPTIDE	C11H20N2O3	2.63E-05	0.00069133	0.45916	2.10E-11	1.63E-09	3.0445	0.0020366	0.015885	1.7947	2.10E-11	1.63E-09	2.3216
54	Caffeine-D3	STIMULANT	C8H10N4O2	NS	NS	NS	0.00031759	0.0013038	1.2387	0.04383	0.038353	1.0007	0.00031759	0.0013038	0.99975
55	L-Carnitine	AMMONIUM COMPOUND	C7H15NO3	0.0043524	0.028653	0.53596	0.018088	0.029062	1.5294	NS	NS	NS	0.018088	0.029062	0.84622

NS- Non-significant
